# Contribution of the NO-GC isoforms to airway responsiveness

**DOI:** 10.1186/2050-6511-16-S1-A67

**Published:** 2015-09-02

**Authors:** Evanthia Mergia, Stefanie Köhler-Bachmann, Michelle Puschkarow, Doris Koesling

**Affiliations:** 1 Institute of Pharmacology, Ruhr-University Bochum, Bochum, Germany; 2 Department of Experimental Pneumology, Ruhr-University Bochum, Bochum, Germany

## 

Hyperreactivity of airways to bronchoconstrictive agents is a common feature of reactive airway diseases. In addition to its established role on vascular smooth muscle tone, the NO/cGMP pathway is also expected to balance the contractile responses of airway smooth muscle. The NO-sensitive guanylyl cyclase (NO-GC) which forms cyclic GMP in response to NO holds a key position in this pathway and exists in two isoforms, NO-GC1 and NO-GC2, which both have been identified in bronchial and pulmonary blood vessels smooth muscle.

Here we determined the contribution of the NO-GC isoforms to the regulation of airway resistance. Airway resistance was determined in a whole body plethysmography chamber in conscious mice deficient in either NO-GC1 or NO-GC2 in response to methacholine and serotonine inhalation. L-NAME was applied to NO-GC KOs to analyse the effect mediated by the remaining NO-GC isoform and to WT to inhibit both isoforms to see a possible synergistic or antagonistic action. The ganglionic blocker hexamethonium was use to differentiate bronchial and neuronal pathways.

Preliminary results indicate that both NO-GC isoforms contribute to airway responsiveness.

